# Neonatal ECMO

**DOI:** 10.3389/fmed.2018.00289

**Published:** 2018-10-25

**Authors:** Cornelia Heleen Van Ommen, Cindy E. Neunert, Meera B. Chitlur

**Affiliations:** ^1^Department of Pediatric Hematology, Sophia Children's Hospital Erasmus MC, Rotterdam, Netherlands; ^2^Department of Pediatrics, Columbia University Medical Center, New York, NY, United States; ^3^Division of Hematology, Oncology, Carmen and Ann Adams Department of Pediatrics, Children's Hospital of Michigan, Wayne State University, Detroit, MI, United States

**Keywords:** neonate, ECMO, anticoagulation, monitoring, complications

## Abstract

Extracorporeal membrane oxygenation (ECMO) is becoming increasingly utilized to manage neonates with cardiac and respiratory failure. The procedure involves extensive anticoagulation in a sick neonate with underlying disease pathology. In addition, the immature hemostatic system in the neonate adds to the complexity of titrating the necessary anticoagulation. This places the infant at greater risk for life threatening hemorrhage and thrombosis. Managing anticoagulation in these infants is extremely challenging and needs the expertise of a physician with a thorough knowledge of the intricacies of developmental hemostasis and limitations of the current laboratory techniques available to manage anticoagulation. This article provides a brief overview of the developing hemostatic system of the neonate and the challenges associated with managing anticoagulation in this vulnerable population of patients.

## Introduction

Extracorporeal membrane oxygenation (ECMO) is a complex form of extracorporeal technology, which is used as rescue therapy in patients with severe respiratory and/or cardiac failure refractory to conventional treatment. Neonatal ECMO was first achieved in 1975 by Dr. Robert Bartlett for a 1 day old with meconium aspiration (1). According to the 2017 Extracorporeal Life Support Organization (ELSO) registry report, ECMO patients now consist of 44.8% neonate, 24.1% pediatric, and 31.1% adult patients with over 40,000 neonates cared for with ECMO worldwide. Despite improvements in technology and increasing clinical practice, hemostatic complications including hemorrhagic and thrombotic events, remain important causes of morbidity and mortality in neonates on ECMO. These complications are the result of exposure of the patient's blood to an artificial circuit as well as underlying patient factors. Anticoagulation is needed to prevent thrombotic complications; however it also has an associated risk of increased hemorrhage. In neonates, management of anticoagulation is particularly challenging due to the developing hemostatic system. Additional risk factors for hemorrhagic complications include the immature germinal matrix in premature born neonates, the development of disseminated intravascular coagulopathy in neonates with sepsis and surgical and procedural interventions, which occur frequently in this patient group. This review focuses on neonates on ECMO and addresses the developing hemostasis, the epidemiology of the hemostatic complications and the monitoring of anticoagulation, along with its challenges, in this specific age group.

## Developmental hemostasis

The exposure of blood to the foreign, non-endothelial materials of the extracorporeal circuit leads to activation of coagulation, fibrinolysis, and acute inflammatory responses, shifting the hemostatic

balance to a hypercoagulable state. Anticoagulation, usually unfractionated heparin, is necessary to maintain patency of the circuit and to reduce thrombotic complications (2). Especially in neonates and young infants on ECMO, it is important to take into account the developing hemostatic system. Although all components of the hemostatic system are present at birth, important differences exist among neonates, infants, and adolescents. “Developmental hemostasis” has significant consequences for the understanding and management of coagulation in neonates and young infants treated with ECMO therapy (3–5).

Platelets are essential for the primary hemostasis. The number and size of platelets are almost identical between neonates and adults (6). Response to agonists, however, is decreased in neonatal platelets compared to adult platelets and platelets from premature infants have even more profound hyporeactivity than term infants (7). Despite this platelet hyporeactivity, healthy term neonates and premature neonates have shorter bleeding times and closure times (using a platelet function analyzer, PFA-100) than adults (8–10). This inconsistency might be explained by higher plasma levels of Von Willebrand Factor (VWF), higher hematocrit, and a higher percentage of large VWF multimers with increased adhesive activity in neonates than in adults (11–13).

Secondary hemostasis consists of the cascade of coagulation factors leading to the formation of insoluble fibrin. All coagulation factors are produced by the liver and endothelial cells of the fetus and measurable in the plasma at about 20 weeks' gestation. The plasma levels slowly increase until birth (12). At birth, plasma levels of most procoagulant factors, including contact activation factors, and vitamin K-dependent factors, are ~50% of normal adult values, which are reflected in the prolonged prothrombin time (PT) and activated partial thromboplastin time (APTT) (13). The levels of many anticoagulant factors, including antithrombin, protein C and S are decreased, as well. During the fibrinolytic process, tissue plasminogen activator (t-PA) and urokinase convert plasminogen into plasmin, which breaks down fibrin, releasing fibrin degradation products, including D-dimer. At birth, plasma levels of most components of the fibrinolytic system differ from older children and adults (14). Plasma plasminogen levels are decreased in neonates.

Despite all the aforementioned differences, healthy neonates have normal hemostasis. Illnesses, however, may easily disturb the hemostatic balance due to lack of reserve capacity, triggering bleeding or thrombotic complications. Neonates with thrombocytopenia, for example, can only increase megakaryocyte number in the bone marrow, whereas adults can increase both megakaryocyte number and megakaryocyte ploidy to improve the number of platelets (15). *In vitro*, transfusion of hyperreactive adult platelets into neonatal blood decreased clotting time, potentially increasing the hypercoagulable state or thrombotic risk in neonates (16). Furthermore, as discussed below the immature neonatal coagulation system may influence the monitoring and efficacy of heparin and use of thrombolytic agents, when needed.

## ECMO use in neonates

Since its first use in 1975 three trials have supported the use of ECMO in the neonatal population (17–19) and as mentioned above a growing number of neonates have been placed on ECMO. Similar to children and adults, neonates may be placed on VV ECMO (diverting blood back to the venous system) or VA ECMO (diverting blood back to the arterial circulation). In addition, neonates may undergo ECPR in which VA ECMO is initiated in the setting of cardiopulmonary arrest. With regards to hemostasis and thrombosis events, VV ECMO has the advantage of pulsatile flow and allows the pulmonary bed to serve as a filter against systemic thrombi. VA ECMO on the other hand, provides non-pusatile flow, requires ligation of the carotid artery, and allows for thrombi within the circuit to enter into systemic circulation (20).

Physiologic criteria for ECMO respiratory support in reversible cardiorespiratory failure include an oxygenation index of >40, PaO_2_/FiO_2_ ratios of < 60–80, a lack of response to mechanical ventilation and other forms of rescue therapy, and elevated ventilator pressures and/or signs of barotrauma. For cardiac ECMO, patients should have evidence of poor oxygen delivery and organ perfusion including hypotension despite use of two medications and low cardiac output with evidence of organ malperfusion, oxygen saturations < 50%, or persistent lactate >4.0. In addition to above, cardiac ECMO is also indicated for pre-operative stabilization, patients who fail to wean from cardiopulmonary bypass during cardiothoracic surgery, and support during high risk catheter procedures (21).

While respiratory failure remains the number one indication for neonatal ECMO (77%), the use of ECMO for cardiac indications is growing (22, 23). The most common respiratory diagnoses were congenital diaphragmatic hernia, meconium aspiration, and pulmonary hypertension (22). With advances in management of neonatal conditions perhaps fewer children will require ECMO for pulmonary failure related to etiologies such as sepsis and ARDS. At the same time ECMO is becoming standard of care over medical management for many neonatal cardiac conditions. While only representing 19% cases overall, 33% of cases between 2009 and 2017 were for a cardiac indication. The most common indication being congenital heart disease with hypoplastic left heart being most common. In a single center report of cardiac indications failure to wean for cardiopulmonary bypass (25%), post-operative cardiac arrest (46%), low cardiac output syndrome (21%), and hypoxemia (7%) were all recognized indications for cardiac ECMO (24).

## Complications of neonatal ECMO

Regardless of the indication for ECMO and/or the type of ECMO that is employed, there is a fine balance between the complications of bleeding and thrombosis. This may be even more intricate in the neonatal population given the complexities of developmental hemostasis outlined above. ELSO defines hemorrhagic events as those resulting in a transfusion of >20 ml/kg/24 h or >3 units/24 h in either the gastrointestinal, pulmonary, CNS, peripheral/mediastinal cannulation site, or surgical sites^1^. The most common hemorrhagic event in neonates is surgical site bleeding (12.4% respiratory and 29.1% cardiac), followed by cannula site bleeding (18.2% respiratory and 10.7% cardiac), and lastly GI (4.2% respiratory and 1.1% cardiac). CNS hemorrhage occurred in 11.5% of neonates with a cardiac indication and 6.4% with a respiratory indication, representing the most common patient-related complication associated with mortality (43 and 25% associated survival). Higher rates of CNS hemorrhage were seen in neonates who underwent ECPR with rates of 14.7%^1^.

In an expanded site specific study among eight institutions rates of site specific bleeding events (defined blood loss requiring transfusion or intracranial hemorrhage) were: surgical site (35.5%), chest tube (30.3%), cannula site (29.6%), pulmonary (9%), gastrointestinal (4.1%), genitourinary (4.1%), intracranial (20.6%). Additionally, the overall estimated bleeding rate for all children was 27.5 events per 100 ECMO days and 2.6 new cases of intracranial hemorrhage per 100 ECMO days (25). Of the neonates who survived to discharge there was no difference in functional status score between infants who experienced a bleeding event compared to those who did not, this however does account for specific types of bleeding such as intracranial hemorrhage which may decrease functional status to a greater degree than other reported bleeding events (25).

Definitions for thrombosis in the ELSO database include those that result in mechanical failure such as thrombosis in the circuit component or those in the hemofilter. According to the ELSO registry, 20% of patients require a circuit change or change of components due to thrombotic issues. The ELSO database only captures two patient- related thrombotic complications, diffuse or localized CNS and limb ischemia. The study by Dalton et al. reported more detailed information on patient-related thrombosis (25). They found that ~12% of neonates experience a patient related thrombotic event (4.5% intracranial infarct, 3.4% limb ischemia, and 0.3% other). No infants suffered an intracardiac thrombosis or pulmonary embolus, and only 2 had thrombosis of the aortopulmonary shunt. A total of 33.7% of neonates had circuit-related thrombotic events in this study. It is possible that thrombotic events are not as appreciated secondary to the fact that infarcts may undergo hemorrhagic conversion and the inability for neonates on ECMO to undergo MRI.

At present ECMO is contraindicated in neonates < 35 weeks due to early reports of increased ICH in this population accompanied by very poor overall survival (26). Since this original report, however ECMO technology and supportive care has increased greatly, perhaps improving outcomes for the most vulnerable population. Despite this concern, compared to infants who were 34 weeks, infants 29–33 had no difference in rates of ICH (17 vs. 21%, *p* = 0.20). Infants in this group did, however demonstrate inferior survival (48 vs. 58%, *p* = 0.01) and higher rates of cerebral infarct (22 vs. 16%, *p* = 0.03) (26). The authors concluded that while statistically significant differences existed with regards to survival and cerebral infarcts they remained clinically acceptable and argued for a lower threshold of 29 weeks. For this group of patients, it is essential to properly review of risks and benefits with family, and maximize medical management (26).

## Prevention and management of hemorrhage and thrombosis in neonatal ECMO

Bleeding events in neonatal ECMO patients are usually multifactorial and can be seen in more than half of the patients. Anticoagulation, surgical site bleeding, consumptive coagulopathy related to the circuit, and underlying disease are primary causes. Management of bleeding differs according to the etiology although common measures may be instituted. First, the dose of anticoagulant can be decreased and/or temporary held irrespective of the cause. Additionally, replacement with blood products (platelets, FFP, cryoprecipitate, and packed red blood cells) should be maximized to normalize coagulation.

Consumptive coagulopathy is a common and often difficult to treat complication of ECMO. Thrombocytopenia occurs due to consumption in the circuit and can be associated with life threatening intracranial hemorrhage. Neonates on ECMO should be closely monitored for this complication with head ultrasounds. In view of the significant morbidity and mortality associated with this complication most centers attempt to maintain platelet counts greater then 100,000/cumm for at least the first 72 h and subsequently will aim to keep the platelet count higher than 50–75,000/cumm. In addition, fibrinogen levels should be monitored and maintained at levels of >100–150 mg/dl routinely using either cryoprecipitate (1 Unit) or FFP (10 cc/kg). The INR should also maintained at < 1.5–2.0. All of these thresholds for blood product replacement have not been adequately investigated and can be adjusted for the consideration of patient complications such as bleeding, extreme fluid overload, and signs of circuit distress. Acquired deficiencies of VWF as well as FXIII have been reported in patients on ECMO and should be considered in cases with severe bleeding despite normal platelet count and normal fibrinogen levels.

In cases of severe life-threatening hemorrhage there are reports of recombinant Factor VIIa being safely used, however the potential benefit must also be weighed against the reports for fatal thrombosis following use (27). While prothromboin complex concentrates have been used for bleeding events in other settings, there is no evidence to support their use in ECMO. The propensity for thrombosis makes this an unlikely product of choice in these patients who are already at an increased risk of thrombosis. Few case reports have described the use of PCC's for the management of intraoperative bleeding during placement on ECMO or cardiopulmonary bypass and one case report describes the fatal massive thrombosis associated with the concomitant use of recombinant FVIIa and PCCs (28). This product would be an option to consider in life threatening episodes of bleeding keeping in mind the significant increase in risk of thrombosis, especially when used in conjunction with recombinant FVIIa. Another approach has been the use of the anti-fibrinolytic agent, aminocaproic acid, to resolve bleeding events and as pre-operative management for congenital diaphragmatic hernia repair. One small study in 29 neonates found aminocaproic acid use to be safe, although it did not appear to influence the incidence of hemorrhagic complications (29). Again further studies are needed to evaluate the safety of adding in a product with potential thrombotic risk. Given that both these agents carry a significant risk of thrombosis, they must be used with extreme caution and should only be used under supervision of a hematologist. In the setting of profound ongoing circuit related coagulopathy and hemolysis, management is limited to changing of components of the circuit or the entire circuit.

Anticoagulation is imperative to prevent circuit and patient- related thrombotic events. Most of the information on the management of anticoagulation in neonates is extrapolated from adult studies. In addition, the target ranges for anticoagulation are based on venous thrombosis studies and have never been validated for ECMO (30). These factors in the context of a very sick child/neonate, a circuit with extensive contact of the blood with artificial surfaces and large doses of heparin results to many issues with monitoring of anticoagulation. The lack of a unified protocol for management of anticoagulation and the variability in circuits and cannulation sites only adds to the potpourri of confusion. Despite, lack of standardized protocol, the primary management is with unfractionated heparin (UFH). UFH has the benefit of a short half-life and being easily reversible with protamine. The efficacy of heparin in neonates might be influenced by the reduced levels of antithrombin. The binding of heparin to antithrombin increases the capability of antithrombin to inactivate especially thrombin and factor Xa by 100- to 1,000-fold. In neonates, low plasma levels of antithrombin may challenge the ability to achieve a sufficient anticoagulant effect using unfractionated heparin resulting in “heparin resistance.” Many centers replace ATIII in an attempt to optimize heparin anticoagulation. This can be accomplished by infusing AT III concentrates (which can be expensive) or FFP (may require large volumes of FFP). The optimal ATIII levels and mode of administration necessary to optimize heparin effect is unknown. Studies have not conclusively demonstrated benefit from AT III supplementation, although some have demonstrated decreased heparin doses with AT III supplementation (31, 32). The effectiveness of antithrombin administration in neonates and young infants on ECMO is unclear.

Use of alternative anticoagulants like Bivalirudin have been reported but the lack of reversal agents, non-specific drug monitoring, and concerns that targeted use of direct thrombin inhibitors will not sufficiently address the amount of thrombin generated by the contact pathway are major limiting factors. Despite this, a potential benefit is that these agents bypass the need for adequate antithrombin and the enormous difficulties with heparin use in the neonatal population.

Occasionally, life-threatening thrombosis can occur on ECMO. Outside of ECMO, thrombolysis is considered for management of such a thrombosis. Systemic TPA is generally contraindicated in neonates due to the high risk of bleeding, and portends an even higher risk of bleeding in neonates on ECMO. Furthermore, TPA may be limited due to decreased plasminogen levels in neonates, as shown in some *in vitro* studies (33, 34). and administration of plasminogen via FFP improved fibrinolysis in cord plasma to adult values (33). While case reports demonstrate successful use (35), this should only be considered after full determination of the risks and benefits in consultation with the family and hematology.

Additional strategies at reducing the complications of ECMO have focused on changes to the circuit itself. As the blood interacts with the surface of the ECMO tubing both the coagulation and complement cascades are activated leading to increased thrombosis risk. Heparin-coated cannula surfaces result is a reduced complement and inflammation reaction, and therefore are likely to reduce thrombosis. Bivalirudin coated surfaces have also been proposed, however these surfaces appear less successful at reducing alternative complement pathway activation compared to heparin. Future directions, include efforts to create true biocompatible surfaces, with similarities to endogenous endothelium (36). Additional cannula characteristics that can influence flow, turbulence, and thrombus formation include catheter length, diameter, use of reprofusion lines, location of cannulation, and side holes (37). All of these may be even more critical when dealing with the rather comparably small vessel size of neonates.

There are also differences in pump dynamics with the two main pump types being roller and centrifugal. Roller pumps come with the added cost of longer tubing which increases the surface area of contact and potential for thrombosis, while concerns exist about higher rates of hemolysis at low flow rates with centrifugal pumps (37). Centrifugal pumps may also allow for passage of micro-emboli not captured by the lung membrane. As of 2011, the majority (85%) of neonatal centers are using roller pumps (37). Changes to the design of centrifugal pumps and low flow oxgenators may mitigate some of these concerns. These differences in pump characteristics, however make comparisons of anticoagulation strategies difficult to directly compare across centers.

## Monitoring anticoagulation in neonatal ECMO

Management of anticoagulation has been controversial in patients on ECLS/ECMO even prior to the broadening of indications for placing neonates on ECMO/ECLS. The armamentarium of tests available to monitor anticoagulation have inched up with time but no significant progress has been made to determine the adequate or ideal test or tests to adequately manage anticoagulation.

The standard tests of coagulation used to monitor UFH, which is still the most commonly used anticoagulant in ECLS/ECMO, include the Activated Clotting time (ACT), aPTT, Unfractionated Heparin Assay (UFH assay), and more recently some centers are also incorporating the newer global assays of coagulation like Thromboelastography (TEG®)/Thromboelastometry (ROTEM®). Each of these has some advantages and disadvantages when used for this purpose. Table 1 provides an overview of the tests currently in use to monitor anticoagulation in neonates and children on ECMO.

**Table 1:** Tests used to monitor anticoagulation on ECMO.

**Test**	**What it measures**	**Factors other than Heparin/anticoagulation affecting the results**	**Recommended target values in uncomplicated ECMO**
ACT	Time for whole blood to clot when activated with kaolin or celite	Coagulation factor deficiency/dysfunction, thrombocytopenia, elevated d-dimers, infection, hypothermia, hemodilution	180 – 220 s
aPTT	Time for recalcified, citrated, platelet poor plasma to clot when activated with an intrinsic pathway activator such as Kaolin. Micronized silica or ellagic acid	Coagulation factor deficiency/dysfunction, hyperlipidemia, hyperbilirubinemia, anti-phospholipid antibody, elevated CRP, hemodilution	50 – 80 s
Anti-Xa	Chromogenic measure of the inhibition of factor Xa by heparin in plasma	Elevated levels of hemoglobin, lipids and bilirubin	0.3 – 0.7 IU/ml
Viscoelastic testing TEG®/ROTEM®	Whole blood assay that measures from clot formation to lysis, using an activator—Kaolin or Tissue factor	Thrombocytopenia, coagulation factor deficiency	TEG® R time: unknown ROTEM® CT INTEM: unknown CT EXTEM: unknown

The ACT is the most widely used test for the monitoring of UFH, and is available at the bedside in real time. Per ELSO guidelines, the ACT is usually titrated to be between 180 and 220 in patients without any bleeding or thrombosis.

The aPTT is the next most common test that is readily available and used for titration of heparin and monitoring of anticoagulation. The baseline aPTT is prolonged in neonates making monitoring using aPTT difficult (38). On the other hand, the recommended target aPTT of 50–80 s can sometimes be a challenge to achieve as both FVIII and Fibrinogen are usually elevated as acute phase reactants in neonates on ECMO.

The Anti-Xa essentially measures the UFH-ATIII complex levels and is more specific to the assessment of heparin effect as it is not affected by abnormalities in other coagulation proteins. It is important to remember that neonates have lower levels of endogenous ATIII. This is especially important as there are 2 types of Anti-Xa assays available commercially. Some where the reagents include excess ATIII to normalize the ATIII (e.g., Berichrom® Heparin) and other assays which does not normalize ATIII (e.g., BIOPHEN Heparin 6®) and may be a better reflection of the inhibition of Factor X by UFH in the neonatal population. Furthermore, younger children and neonates appear to have higher APTT ranges corresponding to therapeutic heparin levels (anti-factor Xa of 0.35–0.70 IU/mL), compared to adults (39). Therefore, the use of anti-factor Xa levels, possibly in combination with APTT, seems to be required in management of unfractionated heparin in neonates and young infants on ECMO.

Global assays of coagulation such as viscoelastic assays (TEG® or ROTEM®), are only recently being explored for use in monitoring anticoagulation in ECMO. These assays measure the viscoelastic properties of the blood and can provide information on clot dynamics and also evaluate fibrinolysis (40, 41). They can be done at the bedside like the ACT but are not available at most centers. The major advantage of these types of assays is the fact that it is the only assay available to evaluate clot strength. While these assays are considered more physiologic, the lack of flow and endothelium in the assay are a major limitation to the claim. In addition, therapeutic ranges for monitoring anticoagulation using TEG/ROTEM have not been established. The Thrombin generation assay (CAT®) is another global assay for studying coagulation and may be considered for evaluation of thrombin generation potential in patients on ECMO. While there is no evidence currently, of the utility of this assay in this situation, there is the theoretical possibility that it may be helpful in assessing the risk of thrombosis or bleeding. This is a plasma based assay, but can be done with or without platelets allowing the ability to evaluate the interaction between cellular and enzymatic factors of coagulation. The major disadvantages are that this is still a research test and the results may not be available in real time by the methods currently in use.

Basic monitoring of anticoagulation should be evaluated given that there are many variables that could affect the measurement of anticoagulant effect and then the results of these various assays should be correlated and linked to the occurrence of bleeding or thrombosis in patients on ECMO.

The ideal assay/combination of assays to monitor anticoagulation on ECMO/ECLS still remains to be determined. Several studies have tried to determine if the aPTT or AntiXa or ACT is the best reflection of heparin anticoagulation (42, 43) but controversies remain. This is a challenge to most physicians managing neonates and children on ECMO. Other studies have also shown no correlation between the results of the coagulation assays and the occurrence of bleeding or thrombosis (25, 44). The addition of the viscoelastic assays to the lab armamentarium has shown some positive correlations although not consistently (45, 46). These assays allow us to evaluate the coagulation status of the patient with and without heparin, therefore allowing us to differentiate an underlying coagulopathy not related to heparin effect itself from surgical causes of bleeding. Some of the inconsistencies in the correlation between routine coagulation assays and heparin dose may be attributed to the effects of plasma free hemoglobin and elevated bilirubin levels on these assays (47), and it becomes very important for the physicians managing the patient to be aware of these effects. Being able to put the lab results in the context of the clinical picture and taking into consideration issues related to the circuit, become critical to the optimal management of these patients. So while we may never be able to define one “ideal coagulation assay,” a “combination of ways to assess coagulation,” may be the best we can do.

## Next steps

Optimizing the circuit to prevent thrombotic complications and eliminating the need for anticoagulation is perhaps the most sought after option. Since contact pathway activation in the ECMO circuit is a major driver for thrombosis, investigators are trying to get a better understanding of the role of Factor XII and Factor XI in thrombosis to determine the safety of inhibiting these factors to prevent thrombosis.

FXII plays an important role in both inflammation and coagulation via bradykinin and thrombin, respectively. While FXII deficiency was previously thought to be a risk factor for thrombosis, a FXII deficient mouse model (FXII^−/−^) shows severely impaired thrombus development in both the arterial as well as venous system, indicating that FXII is necessary for thrombosis. On the other hand these mice also do not have an increased spontaneous or traumatic bleeding, which has since been confirmed in rabbits and baboons as well. Since activation of FXII via contact with the ECMO circuit is associated with increased thrombosis, anti FXII antibody is an attractive option to target thrombotic risk without increasing the risk of bleeding. A fully humanized monoclonal IgG (3F7) to FXII was shown to be effective in preventing thrombosis in the rabbit ECMO model by Larsson et al. (48, 49). FXI like FXII is a contact pathway activator, and its primary substrate is FIX but it can also activate Factors V, VIII and X. FXI deficiency in humans is associated with a bleeding diathesis unlike FXII deficiency. Studies have shown that FXIa activates thrombin which in turn activates Thrombin Activatable Fibrinolysis Inhibitor (TAFI) making the clot resistant to fibrinolysis. Similar to FXIIa^−/−^ mice, FXIa^−/−^ mice are also resistant to thrombosis. Phase 1 studies in humans with Antisense Oligonucleotides (ASO's) have shown decreased plasma levels of FXI with no excessive bleeding. Subsequent phase 2 studies in adults for preventing VTE in patients undergoing total knee arthroplasty, showed that the ASO successfully prevented thrombosis without any increased risk for bleeding, making this a potential attractive option for preventing thrombosis in patients on ECMO circuits as well (50, 51).

Nitric oxide polymers, heparin bonding of the ECMO catheters, and improvement in the oxygenator technology are also promising next steps.

The mortality related to cardiac and/or respiratory failure in neonates has decreased on account of the advancements in technology like ECMO. Although there have been great strides, we have far from eliminated the complications of bleeding and thrombosis related to ECMO. Understanding the pathophysiology of thrombin generation in ECMO, finding optimal ways to monitor anticoagulation and potentially eliminating the need for anticoagulation will be the future, especially for this most vulnerable population.

## Conclusion

There are no standard protocols for anticoagulation in ECMO. There is significant variability in the type of cannulation, the materials that are used to manufacture the components of the circuit, the components (the use of a bladder etc.) that constitute the circuit itself as well as surgical techniques used for cannulation. All these factors play a very important role in the initiation of coagulation and thereby influence the complications associated with ECMO. In addition to all these factors, the availability of laboratory support for the management or monitoring of anticoagulation also differs significant from center to center. This variability results in significant difficulty associated with the development of standardized protocols for the management of ECMO across different centers. Above all, it is important to note that, the assessment of coagulation as it stands today, involves the measurement of “time to initiate coagulation.” The currently available assays (ACT, PT, APTT) do not measure “thrombin” which is the end result of initiation of coagulation nor the final product of the process which is the “clot.” Also, all the current assays are plasma based assays and do not measure the contribution of platelets to the formation of the clot. These are significant limitations in our current ability to monitor anticoagulation and need to be addressed moving forward. The newer global assays like the TEG®/ROTEM® or CAT® may offer alternatives to measuring the thrombin generation capacity in these patients, but need to be studied to determine their real utility in this patient population. Small single center studies with controversial results or studies across different centers with variability in circuits and surgical techniques add to the confusion. Unifying our protocols, starting from circuits and surgery to monitoring of anticoagulation is extremely difficult but will allow us to study this vulnerable population in a better and more efficient manner and will assist us in instituting changes that will ultimately be life saving and decrease complications associated with ECMO, until the time when non-thrombogenic circuits become available.

## Author contributions

CV was responsible for the section on developmental hemostasis and introduction of the manuscript. CN wrote the section on definitions and complications related to ECMO. MC was responsible for selection of the topic and assignment of subtopics for discussion and wrote the section on management of anticoagulation in neonatal ECMO. All authors reviewed, edited, and approved the final manuscript.

### Conflict of interest statement

The authors declare that the research was conducted in the absence of any commercial or financial relationships that could be construed as a potential conflict of interest. The reviewer BRB declared a past co-authorship with one of the authors CHVO to the handling Editor.

## Footnotes

^1^(https://www.elso.org/Portals/0/Files/Reports/2017/International SummaryJanuary 2017.pdf)

## References

[1] Hirschl R Bartlett RH Extracorporeal Membrane Oxygenation (ECMO) support in cardiorespiratory failure. In: Tompkins R editor. Advances in Surgery. Chicago: Medical Publishers (1987) p. 189–211. 3318310

[2] Annich G Adachi I. Anticoagulation for pediatric mechanical circulatory support. Pediatr Crit Care Med. (2013) 14(5 Suppl. 1):S37–42. 10.1097/PCC.0b013e318292dfa7 23735984

[3] Andrew M Paes B Milner R Johnston M Mitchell L Tollefsen DM. Development of the human coagulation system in the healthy premature infant. Blood (1988) 72:1651–7. 3179444

[4] Andrew M Paes B Milner R Johnston M Mitchell L Tollefsen DM. Development of the human coagulation system in the full-term infant. Blood (1987) 70:165–72. 3593964

[5] Manco-Johnson MJ. Development of hemostasis in the fetus. Thrombosis Res. (2005) 115(Suppl. 1):55–63. 15790157

[6] Wiedmeier SE Henry E Sola-Visner MC Christensen RD. Platelet reference ranges for neonates, defined using data from over 47,000 patients in a multihospital healthcare system. J Perinatol. (2009) 29:130–6. 10.1038/jp.2008.141 18818663

[7] Israels SJ. Diagnostic evaluation of platelet function disorders in neonates and children: an update. Semin Thromb Hemost. (2009) 35:181–8. 10.1055/s-0029-1220326 19408191

[8] Andrew M Paes B Bowker J Vegh P. Evaluation of an automated bleeding time device in the newborn. Am J Hematol. (1990) 35:275–7. 10.1002/ajh.2830350411 2239923

[9] Israels SJ Cheang T McMillan-Ward EM Cheang M Evaluation of primary hemostasis in neonates with a new *in vitro* platelet function analyzer. J pediatr. (2001) 1381:116–9. 10.1067/mpd.2001.109794 11148524

[10] Roschitz B Sudi K Kostenberger M Muntean W. Shorter PFA-100 closure times in neonates than in adults: role of red cells, white cells, platelets and von Willebrand factor. Acta Paediatr. (2001) 90:664–70. 10.1111/j.1651-2227.2001.tb02431.x 11440101

[11] Del Vecchio A Latini G Henry E Christensen RD Template bleeding times of 240 neonates born at 24 to 41 weeks gestation. J Perinatol. (2008) 28:427–31. 10.1038/jp.2008.10 18273029

[12] Katz JA Moake JL McPherson PD Weinstein MJ Moise KJ Carpenter RJ. Relationship between human development and disappearance of unusually large von Willebrand factor multimers from plasma. Blood (1989) 73:1851–8. 2785416

[13] Monagle P Barnes C Ignjatovic V Furmedge J Newall F Chan A. Developmental haemostasis. impact for clinical haemostasis laboratories. Thromb Haemost. (2006) 95:362–72. 10.1160/TH05-01-0047 16493500

[14] Albisetti M. The fibrinolytic system in children. Sem. Thromb Hemost. (2003) 29:339–48. 10.1055/s-2003-42585 14517746

[15] Sola-Visner MC Christensen RD Hutson AD Rimsza LM. Megakaryocyte size and concentration in the bone marrow of thrombocytopenic and nonthrombocytopenic neonates. Pediatr Res. (2007) 61:479–84. 10.1203/pdr.0b013e3180332c18 17515875

[16] Ferrer-Marin F Chavda C Lampa M Michelson AD Frelinger AL III Sola-Visner M. Effects of in vitro adult platelet transfusions on neonatal hemostasis. J Thromb Haemost. (2011) 9:1020–8. 10.1111/j.1538-7836.2011.04233.x 21320282 PMC3130591

[17] O'Rourke PP Crone RK Vacanti JP Ware JH Lillehei CW Parad RB. Extracorporeal membrane oxygenation and conventional medical therapy in neonates with persistent pulmonary hypertension of the newborn: a prospective randomized study. Pediatrics (1989) 84:957–63. 2685740

[18] UK collaborative randomised trial of neonatal extracorporeal membrane oxygenation UK Collaborative ECMO Trail Group. Lancet (1996) 348:75–82. 10.1016/S0140-6736(96)04100-1 8676720

[19] Bartlett RH Roloff DW Cornell RG Andrews AF Dillon PW Zwischenberger JB Extracorporeal circulation in neonatal respiratory failure: a prospective randomized study. Pediatrics (1985) 764:479–87. 3900904

[20] Fletcher K Chapman R Keene S. An overview of medical ECMO for neonates. Semin Perinatol. (2018) 42:68–79. 10.1053/j.semperi.2017.12.002 29336834

[21] Bavelloni A Piazzi M Raffini M Faenza I Blalock WL. Prohibitin 2: at a communications crossroads. IUBMB Life (2015) 67:239–54. 10.1002/iub.1366 25904163

[22] Barbaro RP Paden ML Guner YS Raman L Ryerson LM Alexander P. Pediatric extracorporeal life support organization registry international report 2016. ASAIO J. (2017) 63:456–63. 10.1097/MAT.0000000000000603 28557863 PMC5626007

[23] Church JT Kim AC Erickson KM Rana A Drongowski R Hirschl RB. Pushing the boundaries of ECLS: outcomes in < 34 week EGA neonates. J Pediatr Surg. (2017) 52:1810–5. 10.1016/j.jpedsurg.2017.03.054 28365109

[24] Howard TS Kalish BT Wigmore D Nathan M Kulik TJ Kaza AK. Association of extracorporeal membrane oxygenation support adequacy and residual lesions with outcomes in neonates supported after cardiac surgery. Pediatr Crit Care Med. (2016) 17:1045–54. 10.1097/PCC.0000000000000943 27648896

[25] Dalton HJ Reeder R Garcia-Filion P Holubkov R Berg RA Zuppa A. Factors associated with bleeding and thrombosis in children receiving extracorporeal membrane oxygenation. Am J Respir Crit Care Med. (2017) 196:762–71. 10.1164/rccm.201609-1945OC 28328243 PMC5620676

[26] Bui KC LaClair P Vanderkerhove J Bartlett RH. ECMO in premature infants. Review of factors associated with mortality. ASAIO Trans. (1991) 37:54–9. 1854553

[27] Long MT Wagner D Maslach-Hubbard A Pasko DA Baldridge P Annich GM. Safety and efficacy of recombinant activated factor VII for refractory hemorrhage in pediatric patients on extracorporeal membrane oxygenation: a single center review. Perfusion (2014) 29:163–70. 10.1177/0267659113499782 23942787

[28] Bui JD Despotis GD Trulock EP Patterson GA Goodnough LT. Fatal thrombosis after administration of activated prothrombin complex concentrates in a patient supported by extracorporeal membrane oxygenation who had received activated recombinant factor VII. J Thorac Cardiovasc Surg. (2002) 124:852–4. 10.1067/mtc.2002.126038 12324751

[29] Horwitz JR Cofer BR Warner BW Cheu HW Lally KP. A multicenter trial of 6-aminocaproic acid (Amicar) in the prevention of bleeding in infants on ECMO. J Pediatr Surg. (1998) 33:1610–3. 10.1016/S0022-3468(98)90591-7 9856877

[30] Hirsh J Raschke R. Heparin and low-molecular-weight heparin: the seventh ACCP conference on antithrombotic and thrombolytic therapy. Chest (2004) 126(Suppl. 3):188S−203. 10.1378/chest.126.3_suppl.188S 15383472

[31] Niebler RA Christensen M Berens R Wellner H Mikhailov T Tweddell JS. Antithrombin replacement during extracorporeal membrane oxygenation. Artif Organs. (2011) 35:1024–8. 10.1111/j.1525-1594.2011.01384.x 22097980

[32] Stansfield BK Wise L Ham PB III Patel P Parman M Jin C. Outcomes following routine antithrombin III replacement during neonatal extracorporeal membrane oxygenation. J Pediatr Surg. (2017) 52:609–13. 10.1016/j.jpedsurg.2016.10.047 27847121

[33] Andrew M Brooker L Leaker M Paes B Weitz J. Fibrin clot lysis by thrombolytic agents is impaired in newborns due to a low plasminogen concentration. Thromb Haemost. (1992) 68:325–30. 10.1055/s-0038-1656374 1440499

[34] Ries M Zenker M Klinge J Keuper H Harms D. Age-related differences in a clot lysis assay after adding different plasminogen activators in a plasma milieu *in vitro*. J Pediatr Hematol Oncol. (1995) 17:260–4. 10.1097/00043426-199508000-00008 7620925

[35] Garcia A Gander JW Gross ER Reichstein A Sheth SS Stolar CJ. The use of recombinant tissue-type plasminogen activator in a newborn with an intracardiac thrombus developed during extracorporeal membrane oxygenation. J Pediatr Surg. (2011) 46:2021–4. 10.1016/j.jpedsurg.2011.06.039 22008344

[36] Kohler K Valchanov K Nias G Vuylsteke A. ECMO cannula review. Perfusion (2013) 28:114–24. 10.1177/0267659112468014 23257678

[37] Jenks CL Raman L Dalton HJ. Pediatric extracorporeal membrane oxygenation. Crit Care Clin. (2017) 33:825–41. 10.1016/j.ccc.2017.06.005 28887930

[38] Ryerson LM Lequier LL. Anticoagulation management and monitoring during pediatric extracorporeal life support: a review of current issues. Front Pediatr. (2016) 4:67. 10.3389/fped.2016.00067 27446890 PMC4916162

[39] Ignjatovic V Furmedge J Newall F Chan A Berry L Fong C. Age-related differences in heparin response. Thromb Res. (2006) 118:741–5. 10.1016/j.thromres.2005.11.004 16360197

[40] Chitlur M Massicotte MP. The perfect measure of hemostasis: a quest for the holy grail. Thromb Res. (2010) 125:481–2. 10.1016/j.thromres.2009.08.022 19896171

[41] Luddington RJ. Thrombelastography/thromboelastometry. Clin Lab Haematol. (2005) 27:81–90. 10.1111/j.1365-2257.2005.00681.x 15784122

[42] Moynihan K Johnson K Straney L Stocker C Anderson B Venugopal P. Coagulation monitoring correlation with heparin dose in pediatric extracorporeal life support. Perfusion (2017) 32:675–85. 10.1177/0267659117720494 28693359

[43] Sulkowski JP Preston TJ Cooper JN Duffy VL Deans KJ Chicoine LG. Comparison of routine laboratory measures of heparin anticoagulation for neonates on extracorporeal membrane oxygenation. J Extra Corpor Technol. (2014) 46:69–76. 24779122 PMC4557514

[44] Anton-Martin P Journeycake J Modem V Golla S Raman L Tweed J Coagulation profile is not a predictor of acute cerebrovascular events in pediatric extracorporeal membrane oxygenation patients. ASAIO J. (2017) 63:793–801. 10.1097/MAT.0000000000000571 28678046

[45] Nair P Hoechter DJ Buscher H Venkatesh K Whittam S Joseph J. Prospective observational study of hemostatic alterations during adult extracorporeal membrane oxygenation (ECMO) using point-of-care thromboelastometry and platelet aggregometry. J Cardiothorac Vasc Anesth. (2015) 29:288–96. 10.1053/j.jvca.2014.06.006 25655210

[46] Saini A Hartman ME Gage BF Said A Gazit AZ Eghtesady P. Incidence of platelet dysfunction by thromboelastography-platelet mapping in children supported with ECMO: a pilot retrospective study. Front Pediatr. (2015) 3:116. 10.3389/fped.2015.00116 26779465 PMC4702183

[47] Kostousov V Nguyen K Hundalani SG Teruya J. The influence of free hemoglobin and bilirubin on heparin monitoring by activated partial thromboplastin time and anti-Xa assay. Arch Pathol Lab Med. (2014) 138:1503–6. 10.5858/arpa.2013-0572-OA 25357112

[48] Larsson M Rayzman V Nolte MW Nickel KF Bjorkqvist J Jamsa A. A factor XIIa inhibitory antibody provides thromboprotection in extracorporeal circulation without increasing bleeding risk. Sci Transl Med. (2014) 6:222ra17 10.1126/scitranslmed.3006804 24500405

[49] Kenne E Renne T. Factor XII: a drug target for safe interference with thrombosis and inflammation. Drug Discov Today (2014) 19:1459–64. 10.1016/j.drudis.2014.06.024 24993156

[50] Gailani D Bane CE Gruber A. Factor XI and contact activation as targets for antithrombotic therapy. J Thromb Haemost. (2015) 13:1383–95. 10.1111/jth.13005 25976012 PMC4516614

[51] Tillman B Gailani D. Inhibition of factors XI and XII for prevention of thrombosis induced by artificial surfaces. Semin Thromb Hemost. (2018) 44:60–9. 10.1055/s-0037-1603937 28898903 PMC5794506

